# Psychometric Properties of the Short Forms of the Social Interaction Anxiety Scale and the Social Phobia Scale in a Chinese College Sample

**DOI:** 10.3389/fpsyg.2020.02214

**Published:** 2020-10-21

**Authors:** Xueyuan Ouyang, Yan Cai, Dongbo Tu

**Affiliations:** School of Psychology, Jiangxi Normal University, Nanchang, China

**Keywords:** social interaction anxiety, social phobia, item response theory, short form, psychometric properties

## Abstract

This study was carried out to examine the factor structure and psychometric properties of the Social Interaction Anxiety Scale (SIAS) and Social Phobia Scale (SPS) in a sample of 1,001 Chinese college students (male: 34%; female: 66%). Confirmatory factor analysis (CFA) indicated that the two-factor shortened version of the SIAS-6/SPS-6 fit the data well. In addition, the item response theory (IRT) method confirmed the construct and items for the 12 items of the SIAS-6/SPS-6 with satisfactory discrimination, threshold parameters, and test information curve. It was concluded that the factor structure and psychometric properties of the SIAS-6/SPS-6 support their use for such assessment in a Chinese college sample.

## Introduction

Individuals with social anxiety disorder (SAD) show significant and persistent concerns or fears in one or more social or performance situations (American Psychiatric Association [APA], 2013). Most individuals experience social anxiety at some point in their lives, but for those with SAD, these symptoms can have a detrimental impact on their lives (e.g., on their education, career, family relationships, and friendships) (Aderka et al., 2012). There are a variety of symptoms commonly associated with social anxiety (e.g., heart palpitations, blushing, trembling, and avoidance) (Blanco et al., 2001), and the situations during which these symptoms are experienced have often been subdivided into two broad categories (Mattick and Clarke, 1998; Blanco et al., 2001). One of these categories is social interaction (e.g., talking with others or participating at a social party). The other is social performance (e.g., eating, drinking or formal speaking in front of others). The Social Interaction Anxiety Scale (SIAS) and the Social Phobia Scale (SPS) (Mattick and Clarke, 1998) are companion measures that were developed as measures of social anxiety within each of these two broad situational categories and are among the most commonly used tools for assessing social anxiety and the outcome of psychosocial therapy. Since Mattick and Clarke (1998) published their paper in 1998, it has been cited over 2,764 times (Google Scholar, 2019), which means that the SIAS and SPS have had an enormous impact on the research and practice of social anxiety.

Many studies from different populations and countries have demonstrated the satisfactory reliability and validity of these scales. For instance, Mattick and Clarke (1998) were the first to show the coefficient of internal consistency (SIAS: 0.88–0.93; SPS: 0.89–0.94) and test-retest reliability (SIAS: *r* > 0.92; SPS: 0.91–0.93) across 4- and 12-week intervals. Excellent internal consistency (Cronbach’s α) of 0.87 and 0.90 of the Chinese versions of the SIAS and SPS, respectively, has also been reported (Ye et al., 2007).

Although the full scales (i.e., the 19-item SIAS and 20-item SPS) have a good psychometric characteristic, the issue regarding their factor structures has not been fully clarified. Previous studies have analyzed the SIAS and SPS simultaneously and separately and used a series of different sample types in different cultures to determine various factor structures for the SIAS/SPS (Fergus et al., 2012; Peters et al., 2012; Mörtberg et al., 2017; Wong et al., 2019). In addition, as the scales have a total of 39 items, they take approximately 15 to 20 min to administer. They are too long to ever be used in epidemiological research where there is pressure to reduce the respondent burden (Peters et al., 2012). Furthermore, over the last decade several short-version scales of the SIAS and SPS have been developed. Many studies have indicated that the short forms of the SIAS and SPS have similar psychometric properties as those of the full forms (Carleton et al., 2014; Fergus et al., 2014; Le Blanc et al., 2014; Erceg-Hurn and McEvoy, 2018; Sunderland et al., 2019). These findings suggest that either of the short-version scales can be used instead of the full scales to efficiently measure social anxiety.

The purpose of this study is to explore the factor structure of the SIAS and SPS in a Chinese college sample. Some factor models discussed earlier were tested and compared in the context of Chinese culture. According to previous studies (Carleton et al., 2014; Le Blanc et al., 2014; Gomez, 2016; Mörtberg et al., 2017; Erceg-Hurn and McEvoy, 2018; Sunderland et al., 2019; Wong et al., 2019), we expected the SIAS-6/SPS-6 to fit the data best. Ye et al. (2007) employed paper-and-pencil full forms to administer the scales, and the Chinese version had better internal consistency and convergent validity. Most self-reported psychometrics were based on classical test theory (CTT) with internal consistency and construct validity. However, CTT approaches do not provide direct clues for accurate social anxiety symptoms at different points in the range of anxiety severity. Given that the SIAS and SPS are valuable measures for social anxiety, a more thorough analysis of their psychometric properties with the item response theory (IRT) method is warranted. Compared with CTT, IRT can provide more complex information about the psychometric properties of the individual assessment items. As the basis of modern psychometric techniques, IRT approaches can offer estimations of individual latent traits and item characteristics (Pang et al., 2019).

The rest of this article is arranged as follows. First, it will introduce the characteristics of participants and the scales used, as well as a brief review of the scale factor most widely used in previous studies. Second, this study will confirm the best factor model of the SIAS and SPS for Chinese college students. Then, the study will analyze the psychometric characteristics of the scales through the IRT method. Finally, some conclusions and limitations for future work will be provided.

## Materials and Methods

### Participants

The college sample included 1,001 Chinese men (34%) and women (66%), who were citizens of China and voluntarily recruited from universities in Jiangxi Province. In this study, individuals were recruited from universities or the Internet through advertisements, and college participants completed the questionnaire survey online or used the paper questionnaire with a small gift. The final proportion of online and paper questionnaires was 1:4, respectively. The participants were approximately 17 to 23 years old (*Mean* = 19, *SD* = 1.28). The majority of the participants came from the fields of science (69%), and the others came from the fields of liberal arts (31%). The sample consisted of four grades: 43.5% were freshmen, 34.6% were sophomores, 20.7% were juniors, and 1.2% were seniors. There are 397 (39.7%) participants from urban areas and 604 (60.3%) from rural areas. All procedures carried out in studies involving human participants met the institutional ethical standards. All individual participants provided informed consent.

### Measures

#### Social Interaction Anxiety Scale (SIAS) and Social Phobia Scale (SPS)

Social Interaction Anxiety Scale (SIAS) and Social Phobia Scale (SPS) (Mattick and Clarke, 1998). In this study, all the factor structures share the same initial pool from the full-length SIAS and SPS (see Table 1). The SIAS and SPS are companion scales that were designed to measure two related situations of social anxiety and fears. The SIAS is a self-report scale in which each item is rated on a 5-point Likert scale, with values ranging from 0 to 4 (i.e., ranging from “Not at all characteristic or true of me” to “Extremely characteristic or true of me”). In the current study, we used the 19-item SIAS (Mattick and Clarke, 1998), which removes Item 5 “I find it easy to make friends of my own age” from the original 20-item scale unpublished measure developed by Mattick and Clarke in 1989. Similarly, the SPS is also a 20-item self-report scale using the same 5-point scores. The Chinese versions were developed by Ye et al. (2007). The internal consistency, split half reliability and retest reliability of the SIAS (SPS) were 0.874, 0.862, and 0.863 (0.904, 0.865, and 0.849), respectively (Ye et al., 2007). There were no inconsistencies between the translations.

**TABLE 1:** Factor structure of the SIAS and SPS found in previous studies.

Study (*Country*)	Sample (*n*)	Number of factors	Item number
**SIAS**			
Mattick and Clarke (1998) (*Australia*)	Clinical (*243*); Student (*482*)	1	1–19
Olivares et al. (2001) (*Spain*)	Students (*654*)	1	1–20
Ye et al. (2007) (*China*)	Students (*1319*)	1	1–19
Rodebaugh et al. (2006) (*United States*)	Students (*445*)	1	1–17
**SPS**			
Mattick and Clarke (1998) (*Australia*)	Clinical (*243*); Student (*482*)	3	(1)2–6, 8, 13, 15–17, 20; (2)1, 7, 10–11, 18–19;(3)9, 12, 14
Olivares et al. (2001) (*Spain*)	Student (*654*)	1	1–20
Ye et al. (2007) (*China*)	Student (*1319*)	1	1–20
**SIAS and SPS joint structure**			
Safren et al. (1998) (*United States*)	Clinical (*167*)	3	*SPS: (1)1, 2, 4, 8–13, 16–17; (2)3, 5, 7, 18–19;SIAS: 2–4, 5–7, 9–11, 13–20*
Osman et al. (1998) (*United States*)	Student (*200*)	2	*SPS:*1–20; *SIAS*: 1–20
Sakurai et al. (2005) (*Japan*)	Clinical (*149)*	3	*SPS:1–20; SIAS:(l)1–3, 6*, 12–15, 17–19; (2)4, 5, 7–11, 16–20
Carleton et al. (2009) (*Canada*)	Clinical (*355*)	3	*SPS:* (l)4, 6, 8, 13, 16, 17; (2)12, 14, 15;*SIAS*: 6, 9, 14, 15, 18
Heidenreich et al. (2011) (*Germany*)	Clinical ***(577)***	2	*SPS:* 1–20; *SIAS:* 1–20
Kupper and Denollet (2012) (*Netherlands*)	Adults (*1598*)	2	*SPS:* 4–7, 1*2*, 14–16, 18–20; *SIAS:2*, 6, 7, 9, 11, 14–18
Peters et al. (2012) (*Australia*)	Clinical (*902*); Students (*164*)	2	*SPS*: 4, 7–8, 15–17; *SIAS:*2, 4, 6, 8, 10, 13
Fergus et al. (2012) *United States*)	Non-clinical (*469*); Clinical (*145*)	2	*SPS, 4*, 5, 811, 18, *19; SIAS: 3*, 6, 8, 16, 18, 19
De Beurs et al. (2014) (*Holland*)	Clinical (*357*)	4	*SPS:(l)2, 4, 8, 11, 16, 17; (2)3, 5–6, 12–13, 15, 18, 20;(3)1, 7, 9–10, 14*
Wong et al. (2019) (*Australia*)	Clinical (*496*)	2	*SPS:* 1–2 0; *S- SIAS:* 1–17

#### Interaction Anxiousness Scale (IAS)

Interaction Anxiousness Scale (IAS) (Leary, 1983). The IAS contains 15 items with self-statements focusing on subjective feelings of anxiety related to social interactions, and Items 3, 6, 10 and 15 are scored in reverse. Items are rated on a 5-point Likert scale, ranging from 1 (completely uncharacteristic) to 5 (extremely characteristic). Total scores were calculated by adding the responses of each item, where higher scores represent higher levels of social anxiety (Cao et al., 2016). The Chinese version of the IAS has excellent psychometric properties in Chinese colleges (Peng et al., 2004; Cao et al., 2016). Calibration was based on individuals’ scores on the IAS.

### Analyses

#### Factor Structure

CFA was first conducted to assess the fit of the previously demonstrated factor structures and to guide the subsequent analyses. For comparison purposes only, the fit indices for all factor structures of the full scales have been included (Carleton et al., 2014; Wang et al., 2019). To validate the scale’s structure in Chinese college students, CFA with Mplus 7.0 was used to test the previously reported factor structures in different cultures (see Table 1): (a) single factor structures of the SIAS (e.g., Mattick and Clarke, 1998; Olivares et al., 2001; Rodebaugh et al., 2006; Ye et al., 2007) and SPS (Olivares et al., 2001; Ye et al., 2007), (b) three-factor model of the SPS (Mattick and Clarke, 1998), (c) two-factor joint model of the SIAS and SPS (Osman et al., 1998; Carleton et al., 2009; Heidenreich et al., 2011; Fergus et al., 2012; Kupper and Denollet, 2012; Peters et al., 2012; Wong et al., 2019), (d) three-factor joint model of the SIAS and SPS (Safren et al., 1998; Sakurai et al., 2005; Carleton et al., 2009), and (e) four-factor joint model of the SIAS and SPS (De Beurs et al., 2014).

The chi-square, the chi-square/degrees of freedom ratio, root mean square error of approximation (RMSEA), comparative fit index (CFI), Tucker-Lewis index (TLI), and standardized root mean square residual (SRMR) were used to evaluate model fit. For the RMSEA and SRMR, the recommended cutoff values for these indices are close to or lower than 0.06, while for CFI and TLI, these indices are close to or higher than 0.95 (Hu and Bentler, 1999; Brown, 2015; Mörtberg et al., 2017).

#### Reliability and Criterion Validity

The current study investigates both the internal consistency with Cronbach’s *α* and McDonald’s coefficient omega (ω; McDonald, 1999) along with CIs for the total of all subscales and each subscale (Dunn et al., 2013). Cronbach’s *α* and McDonald’s omega are probably the most widely used measures of composite reliability. The reliability was interpreted as follows: <0.6 = insufficient, 0.6 to 0.69 = marginal, 0.7 to 0.79 = acceptable, 0.80 to 0.89 = good, and 0.9 or higher = excellent (Barker et al., 1994). In addition, this study also investigates the criterion validity of the scales by using the IAS as the criterion scale.

#### IRT Analyses

CFA was used to obtain the most suitable factor structure to accomplish the following IRT analyses. Here, the graded response model (GRM; Samejima, 1969) was employed to carry out the IRT analysis by using R software (Version 3.6.1) and the R package mirt (Version 1.30; Clalmers, 2012), including three phases.

First, the GRM was applied to evaluate the SIAS/SPS at the item level. The discrimination parameter and threshold parameters were estimated for each item. The discrimination parameter represents the slope of the item characteristic curve (ICC), and is measured at the steepest point. It also refers to how well an item differentiates among levels of the trait below and above the thresholds for that item. Baker (2001) suggested that values below 0.65 belong to low discrimination, values between 0.65 and 1.34 are moderate, and values above 1.34 are high. This study followed these guidelines.

Second, the discrimination parameter and threshold parameters are used to develop an ICC for each item. The ICCs showed that at the same level, the probability of the trait or ability of different participants obtaining the category score is different, and for a certain trait or ability, the probability of different categories is also different. An ICC can be transformed into an item information curve, indicating the amount of item psychometric information contained at all points along *θ* (Olino et al., 2012).

Third, based on the information concept of IRT, the function of information as the latent variable is called the item information function (IIF, *I*_*i*_(θ)). The sum of the individual item information functions equals the test information function (TIF, *I*(θ)) of the scale (i.e., $I\left(\theta \right)=\sum_{i=1}^{m}I_{i}\left(\theta \right)$, *m* is the test length) (Iwata et al., 2016). The standard error of the measurement (*SE*) is an inverse function of this TIF (i.e., $SE\left(\theta \right)=1/\sqrt{I\left(\theta \right)}$). Greater information reflects greater measurement precision or reliability. It can convert the *SE* into the reliability coefficient for different degrees of latent severity in classic psychometric evaluation (i.e., *reliability*(θ) = 1−*SE*(θ)^2^; Thissen and Wainer, 2001).

## Results

### Factor Structure

The fit indices for different structures via CFA in the Chinese college samples are reported in Table 2. From the results, we can see that the 12-item two-factor model of the SIAS-6/SPS-6 (Peters et al., 2012) had the best fit indices (*χ^2^/df* = 3.05, RMSEA = 0.045, CFI = 0.972, TLI = 0.966, SRMR = 0.030) in the Chinese college sample. More concretely, except for the 12-item two-factor model of the SIAS-6/SPS-6 (Peters et al., 2012), the values of CFI and TLI of other models did not reach 0.95. In particular, the table shows the fit indices for the CFA of the short forms of Peter et al.’s one-factor and two-factor models. Between these models, the two-factor model of the SIAS-6/SPS-6 showed better fit, and was deemed the optimum model.

**TABLE 2:** Confirmatory factor analyses of factors structures of the social phobia scale and the social interaction anxiety scale.

Model	Item	*χ^2^*	*df*	*χ^2^/df*	RMSEA	CFI	TLI	SRMR
Single-factor model, SIAS (e.g., Mattick and Clarke, 1998; Olivares et al., 2001; Ye et al., 2007)	19	703.34	152	4.63	0.060	0.896	0.883	0.043
Single-factor model, SIAS (Rodebaugh et al., 2006)	17	575.22	119	4.83	0.062	0.900	0.885	0.043
Single-factor model, SPS (Olivares et al., 2001; Ye et al., 2007)	20	1391.16	170	8.18	0.085	0.896	0.884	0.043
Three-factor model, SPS (Mattick and Clarke, 1998)	20	1374.76	167	8.23	0.085	0.897	0.883	0.043
Four-factor model, SIAS and SPS (De Beurs et al., 2014)	39	2738.05	696	3.93	0.054	0.885	0.878	0.042
Three-factor model, SIAS and SPS (Sakurai et al., 2005)	39	4067.23	701	5.80	0.069	0.811	0.800	0.073
Three-factor model, SIAS and SPS (Safren et al., 1998)	32	1708.06	461	3.70	0.052	0.909	0.902	0.040
Two factor model, SIAS and SPS (Osman et al., 1998; Heidenreich et al., 2011)	39	2783.72	701	3.97	0.054	0.883	0.876	0.043
Two factor model, SIAS and SPS (Wong et al., 2019)	37	2708.82	628	4.31	0.058	0.869	0.861	0.058
One factor model, SIAS-6/SPS-6 (Peters et al., 2012)	12	657.94	54	12.18	0.106	0.846	0.812	0.075
**Two factor model, SIAS-6/SPS-6 (Peters et al., 2012)**	**12**	**161.53**	**53**	**3.05**	**0.045**	**0.972**	**0.966**	**0.030**
Two factor model, RSIAS/RSPS (Fergus et al., 2012)	12	338.88	53	6.39	0.073	0.932	0.915	0.036
Three-factor model, SIPS (Carleton et al., 2009)	14	367.54	74	4.97	0.063	0.949	0.937	0.035
Two factor model, SIPS (Carleton et al., 2009)	14	375.11	76	4.94	0.063	0.948	0.938	0.036
Two factor model, ASIAS/ASPS (Kupper and Denollet, 2012)	21	972.60	188	5.17	0.065	0.917	0.907	0.038

*RMSEA = Root mean square error of approximation; CFI = Comparative Fit Index; TLI = Tucker Lewis Index; SRMR = Standard root mean square residual. Cut-off values: RMSEA = ≤ 0.06, CFI = ≥ 0.95, TLI = ≥ 0.95, SRMR = ≤ 0.06.*

Based on the results from the Table 2, we further studied the 12-item two-factor model of the SIAS-6/SPS-6 (Peters et al., 2012). The factor loadings of the 12 items are shown in Table 3, and all factor loadings are more than 0.40 (*p* < 0.001) in their corresponding factor. Therefore, the two-factor structure of the SIAS-6/SPS-6 not only has the fewest items but also has the best fit with the Chinese college sample.

**TABLE 3:** Factor loading of items from SIAS-6/SPS-6.

Item	*Mean*	*SD*	SIAS-6	SPS-6
1. I have difficulty making eye contact with others	1.54	1.14	0.63	
2. I find it difficult mixing comfortably with the people I work with.	1.05	0.98	0.60	
3. I tense up if I meet an acquaintance on the street	1.00	1.06	0.59	
4. I feel tense if I am alone with just one person.	1.57	1.11	0.63	
5. I have difficulty talking with other people	1.01	0.93	0.56	
6. I find it difficult to disagree with another’ s point of view	1.82	1.11	0.42	
7. I get nervous that people are staring at me as I walk down the street	1.85	1.27		0.72
8. I worry about shaking or trembling when I’m watched by other people	1.25	1.13		0.75
9. I would get tense if I had to sit facing other people on bus or train	1.34	1.14		0.71
10. I worry I might do something to attract the attention of other people	1.84	1.26		0.68
11. When in an elevator, I am tense if people look at me	1.42	1.25		0.80
12. I can feel conspicuous standing in a line	1.07	1.07		0.69
**The Cronbach’s *α***			**0.742**	**0.868**
**Coefficient omega**			**0.74** CI [0.71,0.77]	**0.87** CI [0.86,0.88]

### Reliability and Criterion Validity

**FIGURE 1:**
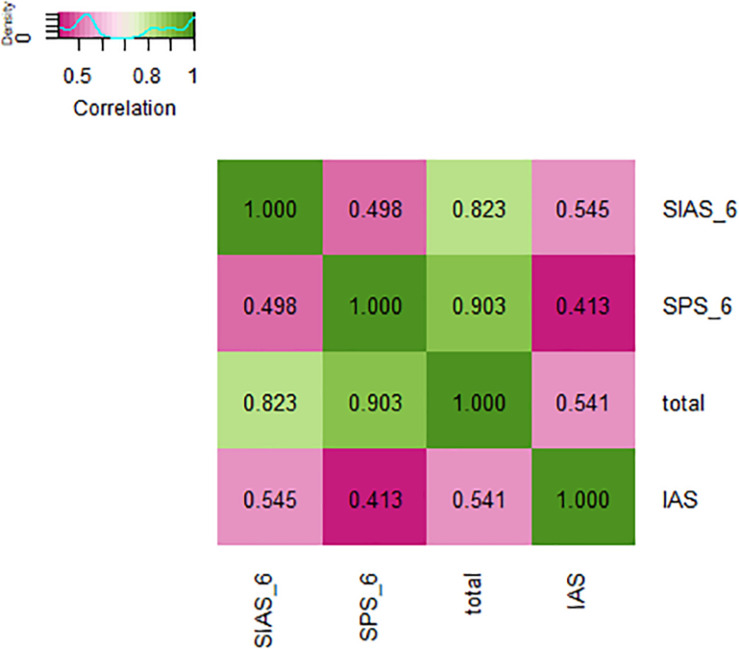
Heatmap of correlation: The values in the grid represent correlation coefficients.

From Table 3, the results show that the Cronbach’s *α* coefficients of the SIAS-6 and SPS-6 are acceptable. The coefficient omega of the SIAS-6 and SPS-6 shows that omega performs at least as well as alpha. Regarding criterion validity, the SIAS-6/SPS-6 score was positively related to the IAS score (*r* = 0.541, *p* < 0.01), and every subscale of the SIAS-6/SPS-6 was also positively related to the IAS score (*r* = 0.545, *p* < 0.01; *r* = 0.431, *p* < 0.01). From Figure 1, the results show that the SIAS-6/SPS-6 has reasonable criterion validity.

### IRT Analyses

Table 4 shows the parameterization of the 12-item scale based on the GRM. It should be noted that item discrimination in the GRM depends on the *a*_*i*_ and the distances among *b*_*ik*_ parameters, so item discrimination can be considered generally adequate.

**TABLE 4:** IRT parameters of the SIAS-6/SPS-6 items.

Item	a *(SE)*	b1 *(SE)*	b2 *(SE)*	b3 *(SE)*	b4 *(SE)*
1	1.71 (0.11)	−1.24 (0.08)	0.30 (0.06)	0.93 (0.07)	2.51 (0.16)
2	1.75 (0.12)	−0.64 (0.06)	1.05 (0.07)	1.67 (0.10)	3.24 (0.22)
3	1.69 (0.12)	−0.38 (0.06)	1.05 (0.07)	1.58 (0.10)	3.01 (0.21)
4	1.65 (0.11)	−1.31 (0.09)	0.18 (0.06)	0.94 (0.07)	2.85 (0.19)
5	1.60 (0.12)	−0.72 (0.07)	1.19 (0.08)	1.92 (0.11)	3.43 (0.24)
6	0.89 (0.08)	−2.60 (0.24)	−0.22 (0.09)	0.90 (0.11)	3.85 (0.37)
7	2.09 (0.12)	−1.18 (0.07)	−0.17 (0.05)	0.54 (0.05)	1.56 (0.09)
8	2.55 (0.15)	−0.60 (0.05)	0.50 (0.05)	1.18 (0.06)	2.03 (0.10)
9	2.23 (0.13)	−0.77 (0.06)	0.44 (0.05)	1.17 (0.07)	2.03 (0.11)
10	1.97 (0.11)	−1.22 (0.08)	−0.13 (0.05)	0.59 (0.05)	1.62 (0.09)
11	3.08 (0.19)	−0.58 (0.05)	0.31 (0.04)	0.88 (0.05)	1.58 (0.08)
12	2.16 (0.13)	−0.38 (0.05)	0.74 (0.05)	1.51 (0.08)	2.39 (0.13)

The item discrimination parameters range from 0.89 to 3.08, and the average value is 1.948 (see Table 4). However, a higher discrimination parameter does not mean that it is a “better” item; both the discrimination parameters and category threshold parameters need to be considered (Rauch et al., 2008). Item location index *b*_*ik*_ represents the level of the latent trait when an individual has a 0.50 chance of picking an option in a given direction (such as a selection that matches a trait). The *b*_*ik*_ parameters of the 12 items ranged from −2.60 to 3.85, showing that they have a wider range coverage of trait values and can be suitable for different levels of anxiety; the location parameters of all the items in this scale are incrementally positive. At the same time, there was no increase or decrease confounding phenomenon, which is consistent with the characteristics of graded response model.

### IRT Characteristic of the SIAS-6/SPS-6 Items

**FIGURE 2:**
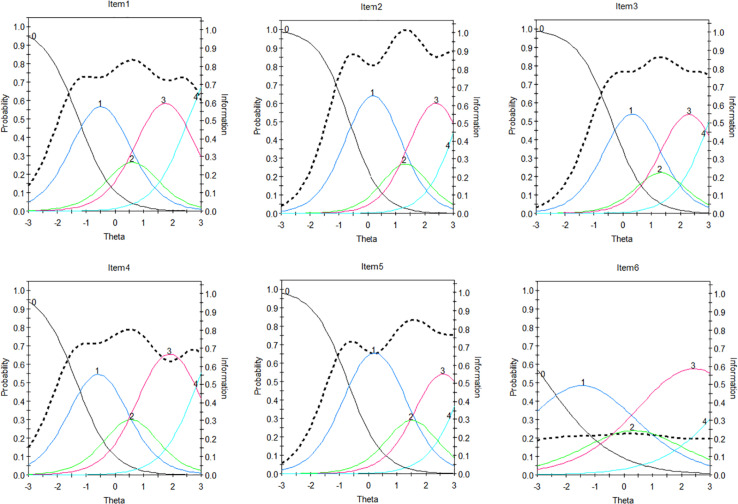
Item Characteristic curves (ICCs) and item information function (IIF) for SIAS-6.

**FIGURE 3:**
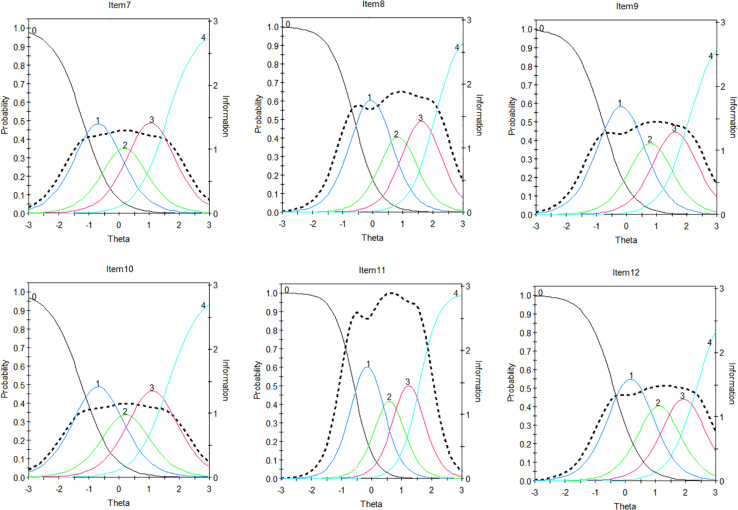
Item Characteristic curves (ICCs) and item information function (IIF) for SPS-6.

To further analyze the characteristics of the items, we draw each item’s characteristic curve and item information curve in Figures 2, 3 (e.g., Item 1…12), respectively. For example, Figure 1 shows that the item has five response categories, ranging from 0 (completely uncharacteristic) to 4 (extremely characteristic) (Mattick and Clarke, 1998). The left-most curve indicates the probability that the individual will select 0 at different trait levels, and this is a monotonic curve, which shows that the lower the trait level is, the greater the probability of choosing 0. The curve on the far right is also a monotonic curve, but it represents the probability of choosing 4 for individuals with different traits. The higher the trait level is, the greater the probability of choosing 4. The curve of each intermediate option presents a single peak shape, which indicates that the probability of selecting the option is the greatest for individuals with only a certain level of the trait.

In the figures, the black dotted line indicates the item information curve. Highly discriminating items have “peaked” information curves because they provide a large amount of information in a narrow range of trait values, whereas low discriminating items have flatter and more spread out information, and as such, they can only provide a small amount of information (Iwata et al., 2016).

Based on the item discrimination parameters, we select 3 items (e.g., Item 2, 6, and 11) as examples for analysis. The discrimination of Item 6 is at the lowest level, that of Item 2 is at the medium level, and that of Item 11 is at the highest level. For Items 2 and 6, the probability of obtaining 2 or 4 points is smaller than that of obtaining other scores. The item information curve of Item 6 is also very low, and there is little information available from this item. This indicates that Item 6 is not good and needs further modifications. The probability of the corresponding score of the participants with different trait levels on Item 11 basically exceeds 0.4, and this item performs well at relatively higher levels of the latent trait, which may involve much information for a whole. This means that the item is good.

### Test Information and Standard Error of Measurement

**FIGURE 4:**
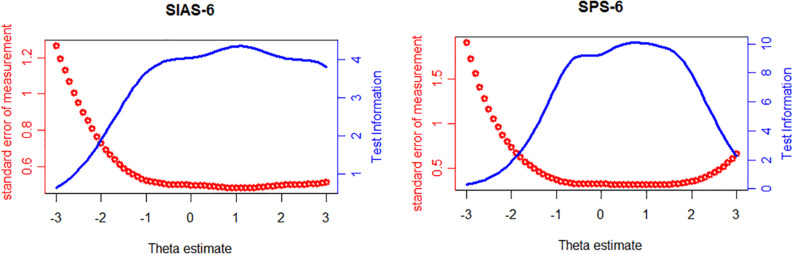
SIAS-6 or SPS-6 information curve and standard error of measurement.

The test information functions and associated standard errors of measurement for the SIAS-6/SPS-6 are displayed in Figure 4. As seen from the left of Figure 4, information was distributed near the average value of the latent trait, with the peak information value at *θ* = 1.1 (information value = 4.35, SE = 0.48). The highest measurement accuracy was from *θ* = −1 to *θ* = 3, where the information values were greater than 0.36, the standard errors were less than 0.524, and their corresponding reliabilities were greater than 0.7. In the right of Figure 4, similar to the SIAS-6, information for the SPS-6 was distributed around the mean of the latent trait, with the peak information value at *θ* = 0.8 (information value = 10.61, SE = 0.31). The range of the highest measurement precision was from *θ* = −1 to *θ* = 2, where the information values were greater than 7.1, the standard errors were less than 0.37, and their corresponding reliabilities were greater than 0.86. These results indicated that the SIAS-6/SPS-6 can provide a great deal of information for most participants and that the quality of the scale is satisfied.

## Discussion

In previous studies, researchers and clinicians used the SIAS and SPS to assess, screen and evaluate treatment outcomes in studying SAD (Clark et al., 2006; Mörtberg et al., 2011, 2017), nevertheless, the culturally validated scale is significant. In this study, the psychometric characteristics of the SIAS/SPS were studied in a Chinese college sample, with the main aim of exploring the factor structure of the scales in the Chinese context.

The factor models suggested in the article with different cultures were tested and compared, and the results showed that the two-factor model of the short form of the SIAS-6/SPS-6 demonstrated acceptable fit. Consistent with most previous studies (Le Blanc et al., 2014; Erceg-Hurn and McEvoy, 2018; Sunderland et al., 2019; Wong et al., 2019), the SIAS-6/SPS-6 was the most widely used short form in the future and exhibited similar psychometric properties as those of the full forms. This means that the two-factor model is robust and has cross-cultural significance. The correlation of the shortened forms with related constructs, such as IAS, shows that the convergent validity of the shortened forms is good.

The IRT analyses revealed encouraging item properties of the SIAS-6/SPS-6. The slope parameters of each item for the SIAS-6/SPS-6 were above 0.89, indicating that each item contributed fully to the test information. The research results of the SIAS-6/SPS-6 on the latent structure of social anxiety indicated that the current Chinese sample shared similar manifest behavior with that of the previous Western research samples. In addition, based on the information provided by the ICC of each item, we identified all the items that performed well in the IRT analyses, but only one to two items could be improved by modification. If an item was either too narrow or too wide, then some options for specific items were combined. For example, in Item 6, options 2 and 3 could be combined into one, or options 1 and 2 could be combined into one, due to the narrow step between thresholds 2 and 3.

However, the current study has some limitations that should be acknowledged. The first is sample characteristics (such as non-clinical samples and clinical samples) which may lead to inconsistency. The findings of the current study were obtained from Chinese college students, and whether they can be generalized to clinical samples or other non-clinical samples (e.g., community adults and senior high school students) requires further validation. The second limitation is about the potency of the interventions for treating SAD. This study did not treat participants so we cannot report on the responsiveness of the short form of the SIAS-6/SPS-6 in a clinical setting. It has been previously shown that the full scale of the SIAS and SPS has good sensitivity to treatment (Acarturk et al., 2009). Whether the short form of the SIAS-6/SPS-6 is as sensitive at detecting changes in social anxiety during treatment as the full scale should be the subject of further study.

## Conclusion

In conclusion, the current study shows that adequate construct validity and excellent psychometric properties support the use of the shortened version of the SIAS-6/SPS-6 in the Chinese contest. Social anxiety is validly captured by the short versions of the SIAS-6/SPS-6, reducing the questionnaire burden for individuals in epidemiological and treatment outcome research.

## Data Availability Statement

The raw data supporting the conclusions of this article will be made available by the authors, without undue reservation.

## Ethics Statement

The studies involving human participants were reviewed and approved by the Moral & Ethics Committee of School of Psychology, Jiangxi Normal University. The patients/participants provided their written informed consent to participate in this study.

## Author Contributions

DT and YC selected the topic and made some modifications of the manuscript. XO collected the data and wrote the manuscript. All authors contributed to the article and approved the submitted version.

## Conflict of Interest

The authors declare that the research was conducted in the absence of any commercial or financial relationships that could be construed as a potential conflict of interest.
